# Molecular Confirmation of G1138A Mutation in FGFR gene in Achondroplasia

**Published:** 2018-06-30

**Authors:** Shyam Bahadur Khanal, Mitesh Shrestha, Hemanta Kumari Chaudhary, Smita Shrestha, Rohit Kumar Pokharel

**Affiliations:** 1 Central Department of Biotechnology, Tribhuvan University, Kirtipur, Kathmandu, Nepal; 2 Nepal Academy of Science and Technology, Khumaltar, Lalitpur, Nepal; 3 Center for Molecular Dynamics- Nepal, Swaraj Sadan, Prasutigriha Marg, Thapathali-11, Kathmandu, Nepal; 4 Institute of Medicine, Maharajgunj Medicine Campus, Tribhuvan University, Maharajgunj, Kathmandu, Nepal

**Keywords:** *achondroplasia*, *dwarfism*, *fibroblast growth factor receptor 3*, *point Mutation*

## Abstract

**Introduction:**

Achondroplasia is the most common form of skeletal dysplasia of genetic origin in humans which is characterized by disproportionate rhizomelic dwarfism. Heterozygous mutation in the transmembrane domain of the FGFR3 gene (4p16.3) occurs as a de novo mutation in most of the cases.

**Methods:**

DNA was isolated from seven samples, out of which, five had clinical features of Achondroplasia while one was dwarf but did not show symptoms of the disorder and one as negative control. PCR was performed for the region incorporating the hotspot region viz. 1138^th^ nucleotide. PCR amplicon of size 164 bp was obtained from all the samples, and was sequenced.

**Results:**

Sequence analysis showed the presence of mutation (G to A transition) in all of the five samples. The five samples that showed the clinical features of Achondroplasia had mutation in the region being analyzed while the single patient who had no clinical manifestations of the disorder despite being dwarf had no such mutation. Among the five patients studied, one patient had a family history of Achondroplasia as observed through pedigree analysis while the remaining four cases were sporadic in nature.

**Conclusions:**

This study further supports that the G1138A mutation is the one of the most common point mutation among Achondroplasia cases. Genetic diagnosis can be useful to identify the disease prenatally and differentiate other life threatening dwarfism for the safety of both mother and fetus.

## INTRODUCTION

Endochondral bone development is negatively regulated by one of the four tyrosine kinases viz. Fibroblast Growth Factor Receptor 3 (FGFR3).^1,2^ Mutations in this gene can develop dysfunctional proteins which impede cartilage growth and development, affect chondrocyte proliferation and calcification.^3^

Among the chondrodysplasias occurring due to gain-of-function mutations in FGFR3 gene, achondroplasia is the most common dwarfism. In achondroplasia, the FGFR3 gene has a point mutation at nucleotide 1138 resulting from either G—A or G—>C.^4^ Approximately 98% of the ACH cases are caused by variation at nucleotide position 1138, with 97% involving a (c1138) G — A mutation and 1% involving a (c1138) G — C mutation.^5,6^

Molecular level diagnosis of Nepalese Achondroplasia is not yet done, thus, in this study FGFR3 gene of five Nepalese ACH cases are analyzed.

## METHODS

The descriptive cross sectional study was conducted in Central Department of Biotechnology of Tribhuvan University, Kathmandu, Nepal. The experiments and the subsequent sequence analysis were done from November, 2017 to January, 2018. Purposing sampling was done. Ethical Approval for conducting the study was obtained from the Institutional Review Board of Institute of Medicine, Tribhuvan University. Individual patient consent was obtained from all of the patients involved in this study. Five clinically diagnosed cases of achondroplasia who consented for the study were included. HochaPudka association of Nepal was approached and approval was taken. Approximately 1-2 ml of whole blood sample was collected from five clinically diagnosed case of achondroplasia, one from an individual with different type of dwarfism and one from normal individual. Blood samples were collected in EDTA vial and transported with cold chain maintenance to Central department of Biotechnology. The samples were stored at 4°C until further analysis.

gDNA was isolated from all blood samples using Quick-g DNA^TM^ Blood Mini Prep kit from ZYMO RESEARCH CORP, USA, dissolved in Tris-EDTA and stored at -20^o^C until required for further use. The extracted DNA was further used for downstream process like Polymerase Chain Reaction (PCR), Restriction Fragment Length Polymorphism (RFLP) and sequencing. Exon 10 on transmembrane domain of the FGFR3 gene was amplified using polymerase chain reaction (^5^prime Thermal Cycler). The sequence of the forward primer was 5′-AGG AGCTGGTGGAGGCTGA-3′, and the sequence of reverse primer was 5′-GGAGATCTTGTGCACGGTGG-3′ (MARCOGEN Company Limited, South Korea). All PCR amplifications were performed in a total of 25 //l volume which contained 12.5 //l of 2 X Master Mix, 0.7 //l of MgCl_2_ (25mM); 2 //l of Template DNA; 1 //l of Forward Primer (100pmol/ //l); 1 //l of Reversed Primer (100pmol/ //l); 8.3 //l of Nuclease free water. Thermal cycling conditions were as follows: Initial denaturation at 95^o^C for 2 minutes, followed by 30 cycles of 95^o^C for 30 seconds, 61 ^o^C for 30 seconds, 72^o^C for 20 seconds, and final elongation at 72^o^C for 5 minutes and storage at 4^o^C for forever. The PCR products were sequenced bi-directionally by Sanger sequencing method (Xcelris Labs Limited; 2^nd^ floor, 22-23 Shrimali Society, Opp. Navrangpura Police Station, Ahmedabad, Gujarat-380015, India) to detect the gene mutation.

The results of sequenced of the PCR product were analyzed with the help of MEGA6 (MEGA 6.06 version) and Clustal Omega (www.clustal.org/omega) and was compared with the normal sequence of the FGFR3 gene obtained from Gene Bank. All mutations were confirmed through forward and reverse sequencing.

## RESULTS

**Figure 1.**
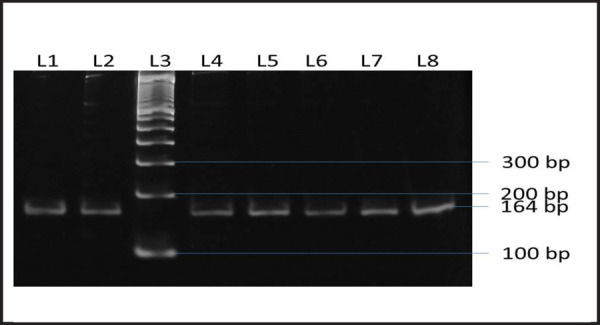
PCR products of Exon 10 of FGFR3 gene region from isolated g-DNA. L1 - Negative Control for Achondroplasia; L2 - pACH003; L3 - 100 bp Ladder; L4 - ACH005; L5 - ACH006; L6 - ACH011; L7 - ACH012; L8 - ACH013.

The mutation hotspot region as previously described by Shianget al. in 1994 was analyzed by PCR using the same set of primers as designated by the authors.^7^ A band size of approximately 164 bps was observed (Figure 1).

**Figure 2.**
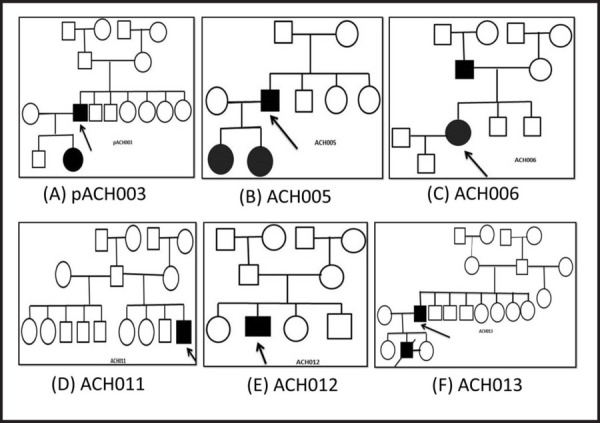
Pedigree Diagram for Samples Collected.

**Figure 3.**
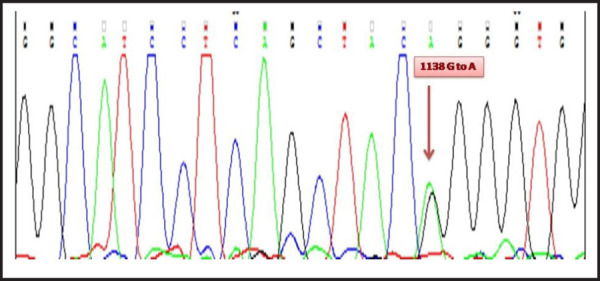
Chromatogram depicting both A and G nucleotide at the heterozygous 1138 nucleotide position.

Patient pACH003, despite suffering from dwarfism, lacked the distinct characteristics of Achondroplastic patients (Figure 2A). This lack of clinical manifestation was further corroborated through sequencing that showed the lack of point mutation at 1138^th^ nucleotide position of FGFR3 gene. For sample ACH005, point mutation at the position of 1138^th^ nucleotide sequence was observed. The heterozygosis at the point of interest could also be observed in the chromatogram, whereby, Guanine was present in one of the allele while Adenine had replaced Guanine in the other (Figure 3). There was no previous history in their family from maternal and paternal sides, hence signaling a sporadic case of Achondroplasia. The mutation was also transferred to the two daughters despite having a normal mother (Figure 2B), representing the typical case of autosomal dominance. Sample ACH006 bore the family history of dwarfism as observed in the pedigree (Figure 2C). Presence of point mutation at the position of 1138^th^ nucleotide sequence confirms our pedigree and is one of the true Achondroplasia cases. The presence of heterozygosis could also be observed from the chromatogram (Figure 3). Here, the father is affected with dwarfism but having married a healthy female was able to give birth to normal phenotypic son. Samples ACH011, ACH012 and ACH013 had no prior family history of Achondroplasia but showed mutation at the 1138^th^ nucleotide position which leads us to the conclusion that the disorder must have occurred due to the sporadic mutation (Figure 2 D, E and F).

## DISCUSSION

As stated by Shiang et al., in 1994 and Pokharel et al., in 1997, true Achondroplasia cases display mutation at the 1138^th^ nucleotide position. The common form of mutation was transition i.e. substitution of Guanine by Adenine. This location of the nucleotide corresponds to the 380^th^ amino acid of the FGFR protein. Due to this missense mutation, the amino acid at the position changes from Glycine to Arginine resulting in protein dysfunction.^10^ Thus produced deficient proteins are incapable of inducing chondrocyte proliferation as well as limits the growth of cartilage and long bones, hence, producing the phenotypic characteristics of Achondroplasia.^11^ Irrespective of race and geography, there is a global agreement that G1138A/C mutation is the cause of the disease in more than 98% of cases.^5,6^ This deduction corresponds with the samples ACH05, ACH06, ACH011, ACH012 and ACH013 as they too display the characteristic features of Achondroplasia and possess the same point mutation as described by the authors.^7^ Four out of five cases had sporadic mutation, one case had inherited the mutation, and all had clinically ACH.

Individual ACH013 was the oldest participant for this study and is living normally at the age of 60 years. The patient has a normal partner and had three children. The first child was normal while the later children were twins, among whom the son was affected and died at the age of 6 years while the daughter was born normal. Individual pACH003 although suffered from dwarfism lacked clinical manifestations for the Achondroplasia disorder and furthermore, sequence analysis showed absence of any point mutation at the position of 1138^th^ nucleotide sequence. However, mutation at position other than 1138^th^ nucleotide might explain the relatively low penetrance of gene and lack of clinical features resembling Achondroplasia patient. Also, the decrease in height may be due to several factors such as lack of nutrition or other underlying genetic predisposition and not due to mutation in FGFR3 gene is also another probability. These are sporadic cases since the parents had normal phenotype up to 3^rd^ generation. Hence, for understanding the actual molecular mechanisms underlying such phenotype, further studies are called for.

For the purpose of maintaining integrity of the research and to ensure that the samples were free of cross contamination, a negative control, blood from a healthy person, was also taken. PCR and sequencing was performed and the result showed no mutation at the designated position. Hence, it can be concluded that the samples were free of contamination and the sequence represent the true data obtained from the samples.

There are several types of dwarfism with different genes involved. Some of which are not compatible to life, like, thanatophoric dysplasia type II osteogenesis imperfect etc.^8^ FGFR3 gene has negative control on the normal development of long bones of body. Mutations at different domains of the gene can cause spectrum of clinical presentation. Intracellular tyrosine kinase domain mutation can cause hypo-achondroplasia, relatively mildest form of dwarfism. However, mutation in the extracellular domain can cause thanatophoric dysplasia, a lethal condition.^8^ These days prenatal ultrasound evaluation of the fetus is a common procedure in antenatal checkup of a pregnant women.^9^ Any growth abnormality of the skeleton can easily be detected prenatally. Prenatal genetic analysis of high risk pregnancy can differentiate lethal and non-lethal conditions. Thus, this simple genetic test can be helpful for prenatal diagnosis of different types of dwarfism, and can give management options for the parents and treating clinicians. The highly prevalent point mutation in the FGFR3 gene might be genetically engineered so that we can treat ACH, a common form of dwarfism.

## CONCLUSIONS

Molecular analysis of Achondroplasia in Nepalese cases has given a similar scenario of sporadic occurrence of ACH due to mutation at 1138^th^ nucleotide of FGFR3 gene found worldwide.
